# Co-circulation of West Nile virus and distinct insect-specific flaviviruses in Turkey

**DOI:** 10.1186/s13071-017-2087-7

**Published:** 2017-03-20

**Authors:** Koray Ergünay, Nadine Litzba, Annika Brinkmann, Filiz Günay, Yasemen Sarıkaya, Sırrı Kar, Serra Örsten, Kerem Öter, Cristina Domingo, Özge Erisoz Kasap, Aykut Özkul, Luke Mitchell, Andreas Nitsche, Bülent Alten, Yvonne-Marie Linton

**Affiliations:** 1 Faculty of Medicine, Department of Medical Microbiology, Virology Unit, Hacettepe University, Ankara, Turkey; 2 Robert Koch Institute, Center for Biological Threats and Special Pathogens 1 (ZBS-1), Berlin, Germany; 3 Faculty of Sciences, Department of Biology, Division of Ecology, Hacettepe University, Ankara, Turkey; 4 Faculty of Arts and Sciences, Department of Biology, Namık Kemal University, Tekirdağ, Turkey; 5 Faculty of Veterinary Medicine, Department of Parasitology, Istanbul University, Istanbul, Turkey; 6 Department of Virology, Faculty of Veterinary Medicine, Ankara, Turkey; 7 Walter Reed Biosystematics Unit, Museum Support Center MRC-534, Smithsonian Institution, Maryland, USA; 8 Department of Entomology, Walter Reed Army Institute of Research, Silver Spring, Maryland, USA; 9 Department of Entomology, National Museum of Natural History, Smithsonian Institution, Washington DC, USA

**Keywords:** West Nile virus, *Flavivirus*, Insect-specific, Biosurveillance, Mosquito, Turkey

## Abstract

**Background:**

Active vector surveillance provides an efficient tool for monitoring the presence or spread of emerging or re-emerging vector-borne viruses. This study was undertaken to investigate the circulation of flaviviruses. Mosquitoes were collected from 58 locations in 10 provinces across the Aegean, Thrace and Mediterranean Anatolian regions of Turkey in 2014 and 2015. Following morphological identification, mosquitoes were pooled and screened by nested and real-time PCR assays. Detected viruses were further characterised by sequencing. Positive pools were inoculated onto cell lines for virus isolation. Next generation sequencing was employed for genomic characterisation of the isolates.

**Results:**

A total of 12,711 mosquito specimens representing 15 species were screened in 594 pools. Eleven pools (2%) were reactive in the virus screening assays. Sequencing revealed West Nile virus (WNV) in one *Culex pipiens* (*s.l.*) pool from Thrace. WNV sequence corresponded to lineage one clade 1a but clustered distinctly from the Turkish prototype isolate. In 10 pools, insect-specific flaviviruses were characterised as *Culex theileri flavivirus* in 5 pools of *Culex theileri* and one pool of *Cx. pipiens* (*s.l*.), *Ochlerotatus caspius flavivirus* in two pools of *Aedes* (*Ochlerotatus*) *caspius*, *Flavivirus* AV-2011 in one pool of *Culiseta annulata*, and an undetermined flavivirus in one pool of *Uranotaenia unguiculata* from the Aegean and Thrace regions. DNA forms or integration of the detected insect-specific flaviviruses were not observed. A virus strain, tentatively named as “*Ochlerotatus caspius flavivirus* Turkey”, was isolated from an *Ae. caspius* pool in C6/36 cells. The viral genome comprised 10,370 nucleotides with a putative polyprotein of 3,385 amino acids that follows the canonical flavivirus polyprotein organisation. Sequence comparisons and phylogenetic analyses revealed the close relationship of this strain with *Ochlerotatus caspius flavivirus* from Portugal and Hanko virus from Finland. Several conserved structural and amino acid motifs were identified.

**Conclusions:**

We identified WNV and several distinct insect-specific flaviviruses during an extensive biosurveillance study of mosquitoes in various regions of Turkey in 2014 and 2015. Ongoing circulation of WNV is revealed, with an unprecedented genetic diversity. A probable replicating form of an insect flavivirus identified only in DNA form was detected.

**Electronic supplementary material:**

The online version of this article (doi:10.1186/s13071-017-2087-7) contains supplementary material, which is available to authorized users.

## Background

Taxonomically distinct viruses transmitted biologically by blood-feeding arthropods to vertebrates are collectively known as arthropod-borne viruses or arboviruses [1]. Mosquitoes, sandflies and ticks serve as frequently-observed vectors that provide a suitable biological environment for propagation and efficient means of access to susceptible species. Arboviruses, particularly those in the genus *Flavivirus* (family *Flaviviridae*) are responsible for some of the endemic and emerging diseases with high human and animal health impact [1, 2].

Flaviviruses are enveloped, single-stranded RNA viruses, comprising over 50 taxonomically recognised species and an increasing number of unclassified or tentative species [3]. Although they share similar genomic and structural properties, flaviviruses display significant variations in susceptible hosts and transmission dynamics. The majority of the flaviviruses have dual hosts and are transmitted horizontally between hematophagous arthropods and vertebrates (tick and mosquito-borne flaviviruses) [3]. Mosquito-borne flaviviruses include the causative agents of the most medically-important human arboviral infections such as dengue virus (DENV), yellow fever virus (YFV) and Japanese encephalitis virus (JEV) [1, 3]. Moreover, some of the emergent arboviruses, including West Nile virus (WNV) and most recently Zika virus (ZIKV), have demonstrated drastic changes in epidemiology and have spread to previously unaffected regions with severe consequences for human populations [4]. Overall, mosquito-borne flaviviruses contribute significantly to the human arboviral disease burden, yet ecological networks of mosquito-borne flaviviruses are varied, complex, and poorly understood [1]. Coupled with the genetic diversity of the viruses, complex mechanisms of pathogenesis and virus-vector-host associations require further study to allow effective prediction and or control of potential or ongoing epidemics [5].

Initially characterised in *Aedes aegypti* cell cultures, insect-specific flaviviruses (ISFs) are phylogenetically distinct from the members of the genus *Flavivirus* and are considered to represent a primordial viral form with replication restricted to insects [6, 7]. ISFs do not replicate in vertebrate cell lines, are not associated with any human or animal disease, and their nomenclature and taxonomic status await official determination [8]. These viruses are globally spread, and several strains have been described from the Americas, Europe and Asia. In Europe, they have been detected in field-collected mosquitoes from Italy, Portugal, Spain, United Kingdom, Czech Republic and Greece. Many ISFs are observed to infect several mosquito species, sometimes belonging to diverse genera encompassing both *Culex* and *Aedes* spp. [8]. Although ISFs are considered to possess the potential to prevent transmission of pathogenic flaviviruses in vectors due to superinfection exclusion or interference, the effects and outcome in natural mosquito habitats is poorly understood [7, 8].

Turkey, located in Asia Minor and Eastern Thrace region of the Balkan Peninsula, forms a transboundary region of the temperate climate zone, connecting Asia, Europe and Africa. The variety of ecological and climatic conditions present throughout the Anatolian Peninsula provide suitable habitats for several mosquito species that can serve as arbovirus vectors [9, 10]. The most widely-studied mosquito-borne flavivirus in Turkey is WNV, for which recent reports have identified a widespread distribution in mosquitoes and infections in several vertebrates, as well as human and equine cases [11–15]. Otherwise, very limited data on mosquito-borne arboviruses is available for Turkey. This study was undertaken to investigate the prevalence and diversity of flaviviruses in mosquitoes, and to provide a risk assessment of the mosquito-borne flaviviruses currently in circulation in Turkey.

## Methods

### Study area, specimen collection and identification

**Fig. 1:**
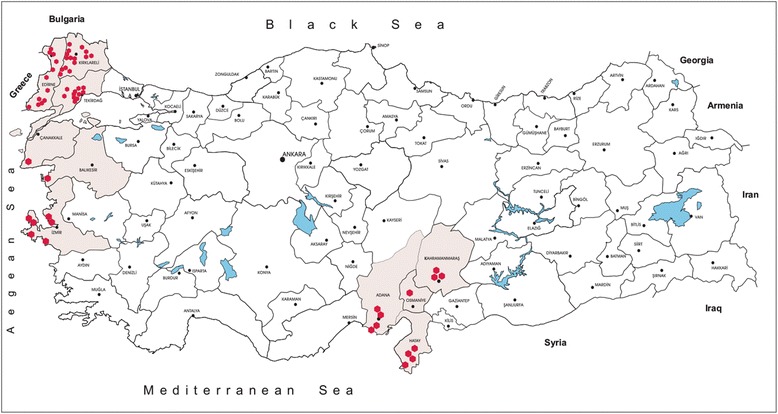
Illustrative map of sampling locations in the study

Mosquito sampling was undertaken between June and October of 2014 and 2015 (Fig. 1) in 58 urban and suburban locations in 10 provinces as follow: Aegean region: Canakkale, Balikesir and Izmir provinces; Thrace region: Edirne, Kirklareli and Tekirdag provinces, Mediterranean and southern Anatolian region: Kahramanmaras, Osmaniye, Hatay and Adana provinces. At each site, standard Miniature Blacklight (UV) traps and CDC Miniature Light traps (John W. Hock Company, Gainesville, FL, USA) were run overnight. In total, 207 traps were deployed overnight and specimens collected the following morningwere immediately transferred on ice. In addition, Hepa filter mouth aspirators and Prokopack aspirator (John W. Hock Company) were utilised for collecting resting adults from inside and outside of nearby human and animal dwellings. All collected specimens were identified to species using available morphological keys [16, 17]. Subsequently, mosquitoes were pooled according to the collection site, species and sex (up to a maximum of 50 individuals per pool) and were stored at −80 °C.

### Mosquito pool processing

Mosquito pools were homogenised by vortexing with 3 mm tungsten carbide beads (QIAgen, Hilden, Germany) in 500–600 μl of Eagle’s minimal essential medium, supplemented with 5% fetal bovine serum, 1% penicillin-streptomycin and 1% l-glutamine. They were clarified by centrifugation at 4,000× *rpm* for 4 min, aliquoted and stored at -80 °C. Nucleic acids were purified from one aliquot of each pool using High Pure Viral Nucleic Acid Kit (Roche Diagnostics, Mannheim, Germany), followed by a reverse transcription reaction, using random hexamers and the RevertAid First Strand cDNA Synthesis Kit (Thermo Fisher Scientific, Tokyo, Japan), performed according to the manufacturers’ guidelines.

### Flavivirus screening

All pools were subjected to nested PCR for the generic detection of flaviviruses. The screening assay utilised is based on degenerated primers targeting the NS5 conserved regions and amplifies all major tick and mosquito-borne pathogenic flaviviruses such as WNV, DENV, YFV, tick-borne encephalitis virus (TBEV), Murray Valley encephalitis virus, Saint Louis encephalitis virus, and Usutu virus, as well as mosquito-specific strains, with a detection limit of 40 TCID_50_ per reaction [18]. PCR products were visualised under ultraviolet light after electrophoresis in 1.5% agarose gels. Negative pools in the NS5 PCR screening were further evaluated using an alternate real-time RT-PCR targeting the same region but with a shorter product [19]. Flavivirus assays were optimised using WNV NY99-4132 isolate, grown on African green monkey (Vero) cells (ATCC- CCL81), and TBEV strain FMSE-H cDNA, obtained from the European Virus Archive (http://www.european-virus-archive.com/). Flavivirus PCR positive pools and cell culture supernatants were further evaluated with the generic nested PCR omitting the cDNA synthesis step, to identify probable DNA forms of the viral genome.

Virus screenings using nucleic acid assays was performed with all necessary precautions and extreme care to prevent carry-over contamination, employing several non-template controls and repeats from the original extracts in the case of a reactive specimen. Pre- and post-PCR steps were strictly performed in spatially-separated areas.

### Sequencing and phylogenetic analysis

Sequencing was carried out in an ABI PRISM 310 Genetic Analyzer (Applied Biosystems, CA, United States) and the resultant chromatograms analysed and aligned using CLC Main Workbench v7.7 (CLCBio, Aarhus, Denmark) and by MEGA software v.6.06 [20]. MEGABLAST, BLASTn and BLASTp algorithms were used for nucleotide and putative protein similarity searches through GenBank (http://blast.ncbi.nlm.nih.gov/Blast.cgi) [21]. Nucleotide and amino acid sequence alignments were generated in CLUSTAL W, implemented in the BioEdit software [22]. Phylogenetic and molecular evolutionary analyses were performed with the maximum likelihood method using Tamura-Nei and Jones-Taylor-Thornton algorithms for nucleotide and amino acid sequences, respectively. These were determined as optimal algorithms using Find best DNA/protein- substitution model tools implemented in MEGA v.6.06.

### Virus isolation and sequencing

Pools positive in the screening assays were inoculated onto semi-confluent monolayers of Vero (ATCC-CCL81) cells and *Aedes albopictus* (C6/36, ATCC-CRL1660) cells, incubated at 37 °C and 28 °C, respectively. Cells were monitored daily for cytopathic effects, and weekly passages to fresh cells were performed. Supernatants were tested for viral nucleic acids using the aforementioned NS5 screening assay.

Culture supernatants with PCR positivity were used for the purification of total RNA, using QIAamp viral RNA extraction kit (QIAgen). Next generation sequencing (NGS) was used for viral genome sequencing. Libraries were prepared using the NexteraXT DNA Library Preparation Kit (Illumina Inc., San Diego, CA, USA), following the manufacturer’s protocols. SuperScript IV Reverse Transcriptase (Thermo Fisher Scientific, Hennigsdorf, Germany) and NEBNext mRNA Second Strand Synthesis Module (New England Biolabs, Frankfurt am Main, Germany) were used for double-stranded cDNA synthesis. The cDNA was cleaned up using Agencourt AMPure XP Reagent (Beckman Coulter Biosciences, Krefeld, Germany) and analysed for yield and size distribution on the Agilent 2100 Bioanalyzer (Agilent Technologies, Waldbronn, Germany). Fragmentation, adaptor ligation and amplification were performed as suggested by the manufacturer. An Illumina HiSeq 1500 (Illumina Inc.) platform was used for the sequencing runs. Reads were aligned to the RefSeq viral nucleotide and protein genome database using MALT (MEGAN alignment tool, v0.3.8) and DIAMOND (v0.7.1) tools [23, 24]. *De novo* assembly of the full genome was carried out in Geneious v9.1 (Biomatters Ltd, Auckland, New Zealand).

## Results

### Distribution and screening of the collected mosquitoes

A total of 12,711 mosquitoes comprising 15 species were collected in Turkey in 2014 and 2015. These included 12,031 specimens (94.7%) from the Thrace region, 440 specimens (3.5%) from the Mediterranean Anatolian region, and 240 specimens (1.9%) from the Aegean region. Overall most abundant was *Culex pipiens* (*s.l.*) (*n* = 5,658; 44.5%), which was the dominant species in all regions (Table 1). *Anopheles maculipennis* (*s.l.*) (*n* = 4,223; 33.2%) was the second most abundant species in both the Mediterranean Anatolian and Thrace regions, whereas *Aedes caspius* (*n* = 2,534; 19.9%), was second most abundant in the Aegean region. The 12 other species identified comprised only 2.3% (*n* = 296) of the entire collection (Table 1). A total of 549 pools were screened in this study: 32 pools (5.8%) from the Aegean region, 44 (8%) pools from the Mediterranean Anatolian region, and 473 pools (86.2%) from the Thrace region (Table 1).

**Table 1:** Distribution of the mosquito specimens according to species, sex and collection region

Species	Aegean			Mediterranean			Thrace			Total	
	♀	♂	Mixed	♀	♂	Mixed	♀	♂	Mixed	No.	%
*Ae. caspius*	76	0	0	25	0	0	2,433	0	0	2,534	19.9
*Ae. geniculatus*	0	0	0	0	0	0	2	11	0	13	0.10
*Ae. pulcritarsis*	1	0	0	0	0	0	0	0	0	1	< 0.01
*Ae. vexans*	0	0	0	0	0	0	2	0	0	2	0.01
*An. claviger*	0	0	0	44	1	0	9	0	0	54	0.4
*An. maculipennis* (*s.l.*)	0	0	0	83	0	0	3,231	0	909	4,223	33.2
*An. superpictus*	1	0	0	0	0	0	0	0	0	1	< 0.01
*Anopheles* spp.	0	0	0	6	0	0	0	0	0	6	0.04
*Cs. annulata*	0	0	0	0	0	0	8	3	0	11	0.08
*Cs. longiareolata*	7	0	0	0	0	0	2	0	0	9	0.07
*Cx. perexiguus*	0	0	0	4	0	0	0	0	0	4	0.03
*Cx. pipiens* (*s.l.*)	107	5	0	171	58	0	3,619	101	1,597	5,658	44.5
*Cx. theileri*	43	0	0	0	0	0	103	0	0	146	1.14
*Cx. tritaeniorhynchus*	0	0	0	48	0	0	0	0	0	48	0.37
*Ur. unguiculata*	0	0	0	0	0	0	1	0	0	1	< 0.01
Total	235	5	0	381	59	0	9,410	115	2,506	12,711	
No. of pools	32			44			473			549	

Eleven of 549 pools (2.0%) were positive in the nested and real-time generic flavivirus PCR assays (Table 2). All pools initially testing negative in the nested PCR remained negative when subjected to the real-time PCR assay. The nested PCR performed without the cDNA step was negative in all pools. Positive pools originated from 7 distinct collection sites in Canakkale (Aegean region) and Kirklareli (Thrace region) provinces, collected between June and November 2014 and June and August 2015. Positive pools comprised *Cx. theileri* (5/11), *Cx. pipiens* (*s.l.*) (2/11), *Ae. caspius* (2/11), *Culiseta annulata* (1/11) and *Uranotaenia unguiculata* (1/11) (Table 2). The overall minimal infection rate (MIR), expressed as the number of positive pools per 1,000 mosquitoes, was calculated as 0.86.

**Table 2:** Features of the flavivirus-positive mosquito pools

Pool code	Region: Site	Elevation (m)	Coordinates	Date	Pool size	Species	Virus detected
T1	Aegean: Canakkale	280	39°29'36.1"N, 26°19'26.7"E	08/2015	20	*Cx. theileri*	*Culex theileri flavivirus*
T2	Aegean: Canakkale	280	39°29'36.1"N, 26°19'26.7"E	08/2015	20	*Cx. theileri*	*Culex theileri flavivirus*
T3	Thrace: Kirklareli	80	41°36'48.6"N, 26°57'56.3"E	08/2015	43	*Cx. theileri*	*Culex theileri flavivirus*
T4	Thrace: Kirklareli	80	41°36'48.3"N, 26°57'56.5"E	11/2014	21	*Cx. pipiens* (*s.l*.)	*Culex theileri flavivirus*
T5	Thrace: Edirne	206	41°48'53.7"N, 26°49'00.7"E	10/2014	1	*Ur. unguiculata*	Unknown *Flavivirus*
T6	Thrace: Kirklareli	80	41°36'48.6"N, 26°57'56.3"E	07/2014	52	*Ae. caspius*	*Ochlerotatus caspius flavivirus*
T7	Thrace: Kirklareli	120	41°40'23.2"N, 26°58'51.1"E	06/2014	41	*Ae. caspius*	*Ochlerotatus caspius flavivirus*
T8	Thrace: Kirklareli	80	41°36'48.3"N, 26°57'56.5"E	10/2014	24	*Cx.pipiens* (*s.l*.)	West Nile virus
T9	Thrace: Kirklareli	317	41°36'48.6"N, 26°57'56.3"E	08/2014	7	*Cx. theileri*	*Culex theileri flavivirus*
T10	Thrace: Kirklareli	317	41°51'48.3"N, 27°00'59.2"E	08/2014	1	*Cx. theileri*	*Culex theileri flavivirus*
T11	Thrace; Kirklareli	112	41°38'12.4"N, 27°10'36.3"E	07/2014	3	*Cs. annulata* (♂)	*Flavivirus* AV-2011

### Analysis and characterisation of partial virus sequences

**Fig. 2:**
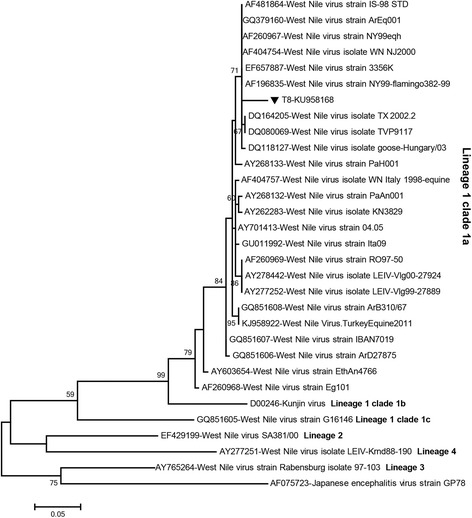
The maximum likelihood analysis of the partial West Nile virus (WNV) NS5 nucleotide sequence (292 bp). The evolutionary distances were computed using the Tamura-Nei model and for 1,000 bootstrap replicates. The sequence characterised in the study is indicated with a reverse triangle, pool code and GenBank accession number. Global virus strains are indicated by GenBank accession number, virus abbreviation and strain/isolate name. Japanese encephalitis virus strain GP78 was included as an outlier

Among the generic flavivirus PCR positive pools, WNV (GenBank accession: KU958168) was characterised from one pool (T8) comprising 24 *Cx. pipiens* (*s.l.*) mosquitoes collected from Kirklareli province (Thrace region) (Table 2). Maximum likelihood analysis placed this sequence among WNV lineage 1 clade 1a strains (Fig. 2), with nucleotide identities of 92.8–97.3% to several global isolates. This partial NS5 sequence demonstrated a 95.6% similarity to the WNV strain characterised in equines from Central Anatolia, Turkey in 2011 (GenBank accession KJ958922 West Nile VirusTurkeyEquine2011) [15]. Interestingly, the original WNV sequence isolates from Turkey remained distinct in the maximum likelihood tree, with our T8 pool sequence (GenBank accession: KU958168) clustering instead with WNV strains from United States, Argentina, Israel and Hungary (Fig. 2).

**Fig. 3:**
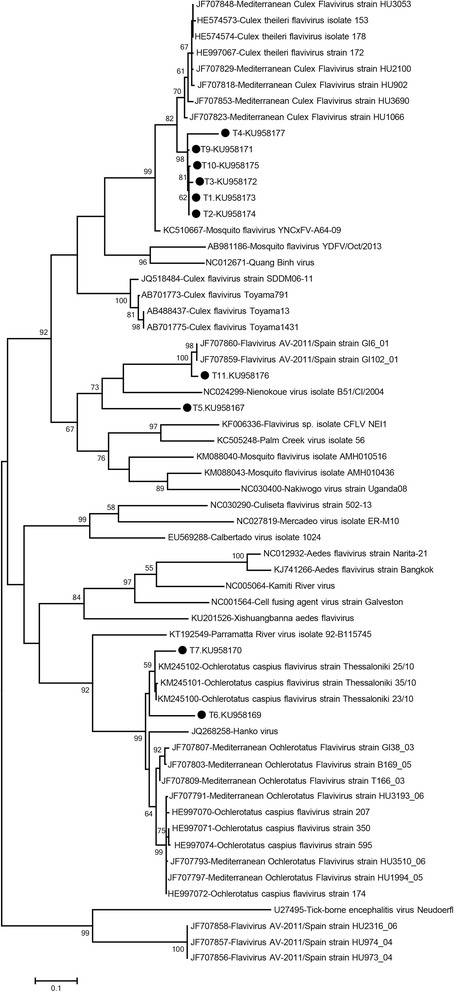
The maximum likelihood analysis of the partial NS5 nucleotide sequences of the insect-specific flaviviruses (421 bp). The evolutionary distances were computed using the Tamura-Nei model and for 1,000 bootstrap replicates. The sequences characterised in the study is indicated with a circle, pool code and GenBank accession number. Virus strains are indicated by GenBank accession number, virus and strain/isolate names as available. Tick-borne encephalitis virus strain Neudoerfl was included as an outlier

**Fig. 4:**
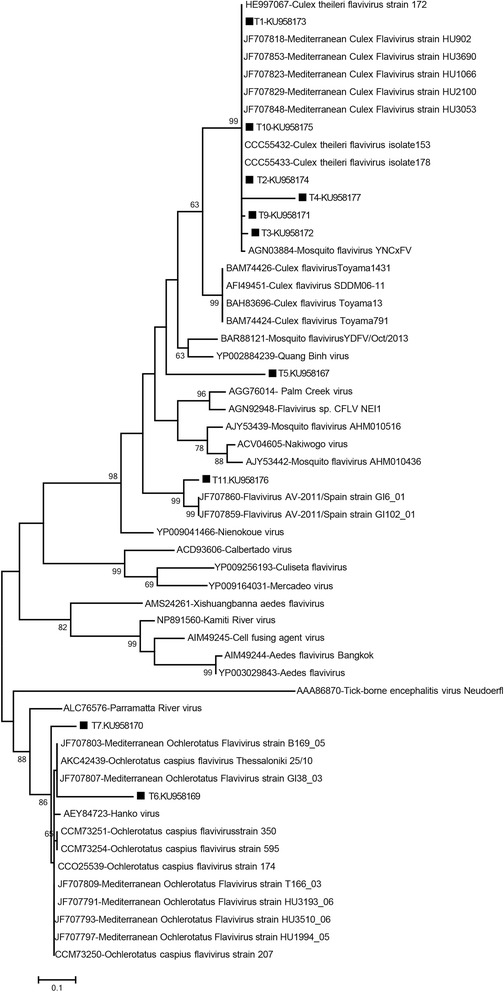
The maximum likelihood analysis of the partial NS5 amino acid sequences of the insect-specific flaviviruses (125 amino acids). The evolutionary distances were computed using the Jones-Taylor-Thornton model and for 1,000 bootstrap replicates. The sequences characterised in the study is indicated with a square, pool code and GenBank accession number. Virus strains are indicated by GenBank accession number, virus and strain/isolate names as available. Tick-borne encephalitis virus strain Neudoerfl was included as an outlier

In 10 of the 11 generic flavivirus PCR positive pools, sequences presumably identified as ISFs were characterised (GenBank accessions KU958167, KU958169–KU958177). The sequences comprise 421–929 nucleotides and correspond to positions 8,915 to 9,844 on the *Culex flavivirus* Toyama791 strain genome (GenBank accession AB701773). Maximum likelihood analysis of these sequences and their putative amino acid sequences revealed the presence of at least three distinct ISFs (Figs. 3 and 4).

All sequences identified in *Cx. theileri and Cx. pipiens* (*s.l.*) pools (T1-T4, T9 and T10; Table 2) demonstrated intramural nucleotide identity rates of 90.6–99.2%. Although they formed a distinct group supported with high bootstrap values in the nucleotide-based tree (Fig. 3), this could not be reproduced in the amino acid-based analysis where the sequences clustered along with *Culex theileri* flaviviruses (CTFVs) and Mediterranean *Culex* flaviviruses (Fig. 4). Nucleotide divergence rates of 5.7–11.5% and 4.9–11.9% were noted in comparison with CTFVs (GenBank accessions HE574573, HE574574, HE997067) and several Mediterranean *Culex* flaviviruses deposited in GenBank, respectively.

Sequences from the T6 and T7 pools detected in *Ae. caspius*, grouped with several Mediterranean *Ochlerotatus caspius* flaviviruses (Figs. 3 and 4). Nucleotide divergence rates of 1.4–13.7% were noted between these sequences and *Ochlerotatus caspius flavivirus* (OCFV) and Mediterranean *Ochlerotatus* flaviviruses included in the analysis.

The viral sequence eminating from pool T11 (*Cs. annulata*: GenBank accession KU958176) demonstrated highest nucleotide similarities to NS5-like sequences from *Flavivirus* AV-2011 strains GI6_01 and GI102_01 from Spain (GenBank accessions JF707859 and JF707860) in MEGABLAST and BLASTn searches. Pairwise comparisons demonstrated nucleotide similarities exceeding 97%. In the maximum likelihood analyses, the T11 pool sequence clustered with, but remained distinct from, these sequences in both nucleotide and amino acid-based trees (Figs. 3 and 4). A separate group of *Flavivirus* AV-2011 sequences (GenBank accessions: JF707856–58), which are divergent from all other ISF sequences, were also included in the analysis (Fig. 3). These contained several internal stop codons and were therefore not included in the maximum likelihood analysis of the partial amino acid sequences (Fig. 4). A reliable viral identification could not be attained for the sequence recovered from pool T5 (*Ur. unguiculata*; GenBank accession KU958167); MEGABLAST could not provide any specific matches and BLASTn displayed limited similarities (< 72%) to several other ISFs. Phylogenetic analyses also resulted in the ambiguous placement of this sequence (Figs. 3 and 4).

### Flavivirus isolation and characterisation in mosquito pools

Homogenates from the flavivirus PCR-positive pools were inoculated onto C6/36 and Vero cells. After five blind passages, no evidence of viral replication was detected in pools T4, T5, T7, T8, T10 and T11, with additional negative flavivirus PCR results in culture supernatants tested after each passage. In 5 pools (T1, T2, T3, T6 and T9), a very mild CPE, characterised by rounding and detachment was noted in *Ae. aegypti* C6/36 cells in 2-4th passages. These supernatants were positive in the screening PCR. No CPE or PCR amplification was observed in mammalian Vero cells inoculated with any of these pools. The PCRs performed without reverse transcription were negative in all supernatants. The virus strain detected in T1, T2, T3 and T9 homogenates was characterised as *Culex theileri flavivirus* (CTFV) and has been reported previously [25]. The NGS performed on the T6 supernatant (passage 4) provided 1,668,342 reads of 250 basepairs. They were trimmed for quality (Phred quality score > 33 with > 99.9% base call accuracy) and nucleotide- and protein-aligned against the complete NCBI viral database, using the MALT and DIAMOND softwares. A total of 88,940 reads were mapped to *Ochleratus caspius flavivirus*, with a coverage of 100%. The obtained sequence was submitted to GenBank under accession number KY345399.

### Analysis of the ISF genome and the putative coding region

The ISF genome comprised 10,370 nucleotides with a 10,158 bp polyprotein-coding region, flanked by 5' and 3' ends of 64 and 148 nucleotides, respectively. MEGABLAST and BLASTn searches revealed highest identity matches (coverage > 90%) to three distinct ISF strains: *Ochlerotatus caspius flavivirus* (OCFV) isolated in Portugal [26]; Hanko virus (HANKV) isolated in Finland [27]; and Parramatta River virus (PARV) isolated in Australia [28]. Alignment and pairwise comparisons of the polyprotein coding region of these isolates demonstrated similarity rates of 67.3–94.7% and 71.9–98.6% on nucleotide and putative amino acid levels, respectively (Additional file 1: Table S1). The isolated ISF is tentatively named as the "*Ochlerotatus caspius flavivirus* Turkey" (OCFVt), based on the nucleotide and amino acid sequence similarities.

**Fig. 5:**
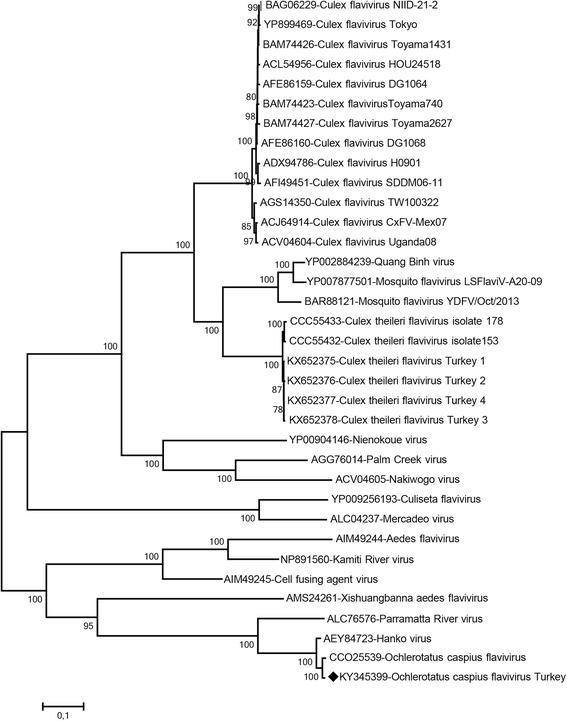
The maximum likelihood analysis of the complete flavivirus polyprotein sequence of the *Ochlerotatus caspius flavivirus* isolated in the study. The evolutionary distances were computed using the Jones-Taylor-Thornton model and for 1,000 bootstrap replicates. The sequence characterised in the study is indicated with a diamond, pool code and GenBank accession number. Virus strains are indicated by GenBank accession number, virus and strain/isolate names

Phylogenetic relationship of the OCFVt isolate with several distinct ISFs was determined. Maximum likelihood analysis of the near-complete flavivirus polyprotein sequences placed OCFVt, OCFV and HANKV in a monophyletic group, that shares a common ancestor with PARV. Nevertheless, OCFVt and OCFV remained phylogenetically distinct, supported with high bootstrap values (Fig. 5)

A ribosomal frameshifting site, comprising a heptanucleotide motif followed by a 5–9 nucleotide spacer region and a pseudoknot or stem-loop structure, was predicted to occur in the majority of the ISFs [7]. This motif is identified encompassing the 3,397–3,403 nucleotides of the OCFVt genome, which is identical to that of HANKV [7].

The putative OCFVt polyprotein comprised 3,385 amino acids (Table 3) and complies with the canonical flavivirus genome organisation with structural proteins virion C-anchored C (C-AC), premembrane-membrane (PrM-M), envelope (E), and non-structural proteins (NS1, NS2a, NS2b, NS3, NS4a, NS4b and NS5) in the given order [3]. Pairwise comparisons with the related ISF strains demonstrated identical or similar polyprotein sizes and 71.9–98.6% amino acid similarities (Additional file 1: Table S1). Region-specific comparisons revealed significant similarities between viral proteins, ranging between 95.6–100% for OCFV and 93.4–99.3% for HANKV (Table 3). A list of the amino acid variations observed among OCFVt, OCFV and HANKV is provided in Additional file 2: Table S2.

**Table 3:** Comparison of the putative amino acid sequences of the *Ochlerotatus caspius flavivirus* Turkey (OCFVt) with other mosquito-specific or mosquito-borne flaviviruses

	OCFVt	OCFV		HANKV		PARV		CTFV153		CxFTokyo		QBV		CFAV		WNVT2	
	Size	Size	ID%	Size	ID%	Size	ID%	Size	ID%	Size	ID%	Size	ID%	Size	ID%	Size	ID%
C + AC	137	137	95.6	137	93.4	140	42.5	136	18.7	139	20.6	136	21.9	136	19.7	123	18.8
PrM + M	147	147	99.3	147	93.8	147	61.9	142	34.4	143	33.1	142	36.4	142	40.5	167	13.7
E	431	431	98.3	431	95.8	431	70.9	427	45.2	427	46.8	427	44.3	427	46.4	501	17.3
NS1	392	392	98.9	392	97.9	396	75.0	369	42.8	369	42.5	369	43.5	390	46.5	352	21.8
NS2a	214	214	99.5	214	96.2	212	61.6	228	17.1	230	20.9	229	20.1	232	22.6	231	14.1
NS2b	149	149	100	149	99.3	149	57.7	143	18.7	142	20.8	143	17.4	124	18.1	131	14.7
NS3	600	600	99.3	600	97.5	599	73.5	577	39.8	578	39.1	578	39.1	577	45.1	619	30.5
NS4a	161	161	98.7	161	96.2	161	64.5	189	16.4	189	17.1	188	17.4	145	17.7	126	8.6
NS4b	260	260	96.9	260	96.9	259	67.4	257	15.6	257	19.8	258	14.8	280	20.2	278	11.8
NS5	894	789	98.7^a^	894	98.2	890	83.3	889	60.7	889	60.1	889	61.7	888	59.2	905	44.1
ORF	3,385			3,385		3,384		3,357		3,363		3,359		3,341		3,433	

*Abbreviations*: *OCFVt Ochlerotatus caspius flavivirus* Turkey (KY345399); *OCFV Ochlerotatus caspius flavivirus* (HF548540); *HANKV* Hanko virus (JQ268258); *PARV* Parramatta River virus isolate 92-B115745 (KT192549); *CTFV153 Culex theileri flavivirus* isolate 153 (HE574573); *CxFTokyo Culex flavivirus* strain Tokyo (AB262759); *QBV* Quang Binh virus (NC012671); *CFAV* Cell fusion agent virus (NC001564); *WNVT2* West Nile virus strain T2 from Turkey (KJ958922)

^a^Based on the 789 amino acid partial sequence available for this strain

Several conserved amino acid motifs observed in flaviviruses were identified in OCFVt polyprotein. Flavivirus glycoprotein central and dimerisation domains (PFAM-ID: PF00869) were identified in E protein (residues: 318–442). The 14-amino acid motif involved in endosomal fusion and cellular entry was present as NRGWGTGCFKWGIG, located in 377–391 amino acids of the OCFVt polyprotein [29]. This motif was identical in OCFV, HANKV, PARV and CTFVs isolated in Portugal and Turkey [25–28]. Flavivirus non-structural protein 1 domain (PFAM-ID: PF00948) was detected (residues: 889–1,074). In NS3, peptidase S7-flavivirus NS3 serine protease (PFAM-ID: PF00949; residues 1,495–1,624), Helicase conserved C-terminal domain (PFAM-ID: PF00271, residues 1,828–1,929), Flavivirus DEAD domain (PFAM-ID: PF07652; residues 1,666–1,803) were identified. Furthermore, ATP (residues: 1,672–1,676) and ion binding (residues: 1,759–1,762) sites, conserved among OCFVt, OCFV and HANKV were noted. Finally, NS5 included the FtsJ-like methyltransferase (PFAM-ID: PF001728; residues 2,533–2,705) and the Flavivirus RNA-dependent RNA polymerase (PFAM-ID: PF00972; residues 2,736–3,377) motifs. The predicted protease cleavage sites are identical to OCFV and HANKV, and the PrM-M junction contains the minimal furin cleavage site, similar to HANKV and Nakiwogo virus [7, 26].

## Discussion

The genus *Flavivirus* is surprisingly diverse within the *Flaviviridae*, including viruses with drastically different transmission patterns, hosts and health impact [7]. The best-recognised flaviviruses circulate between hematophagous arthropods and vertebrates and include prominent mosquito and tick-borne pathogens such as WNV and TBEV. Moreover, several flaviviruses replicating exclusively in insects or vertebrates have been characterised [7, 8]. Vector surveillance programs have been widely undertaken for monitoring the circulation of arthropod-borne flaviviruses important for human or animal health [1, 2]. They further provide information on epidemiology, dispersion, associations with different arthropod species and facilitate characterisation of novel viruses. Such efforts have been rare in Turkey, where geographical and climatic conditions favour the introduction and establishment of arboviruses [10, 11]. This study was undertaken to fill the information gap and provide an update on flavivirus circulation in mosquitoes from geographically distinct regions of Turkey. A total of 12,711 mosquitoes belonging to 15 species and collected from 58 locations in the Aegean, Thrace, and Mediterranean Anatolian regions were evaluated (Table 1), making this the most extensive mosquito arboviral biosurveillance study performed in Turkey to date.

We detected West Nile virus, phylogenetically related to WNV lineage 1 clade 1a strains, in a single pool of *Cx. pipiens* (*s.l.*) mosquitoes collected in the Thrace region (Table 2, Fig. 2). WNV is arguably the most deeply-investigated mosquito-borne arbovirus in Turkey and widespread occurrence of virus exposure as well as symptomatic human and equine infections have been reported [9, 11–15]. Despite the lack of WNV virus detection in specimens from the south-east and central Anatolian campaigns [30, 31], we previously detected in *Cx. pipiens* (*s.s.*), *Ae. caspius*, *Cx. quinquefasciatus* and *Cx. perexiguus* specimens from the Mediterranean Anatolia and Thrace regions, also revealing the presence of *Cx. quinquefasciatus* in Turkey for the first time [11, 12]. In 2012, higher rates of WNV infection in *Ae. caspius* (15.6%) and *Cx. pipiens* (*s.l.*) pools (36.3%) was noted in specimens collected from Thrace [11]. In Europe, *Cx. pipiens* (*s.l*.), *Cx. theileri*, *Cx. modestus*, *Cx. univittatus*, *Ae. caspius* and *An. maculipennis* (*s.l.*) were reported to be infected with WNV and suggested to participate in virus propagation and transmission [32]. In Turkey, the prototype WNV strain was isolated and characterised from an infected horse in 2011 [14, 15]. Interestingly, comparison of the partial NS5 sequences identified in this study with the original Turkish WNV isolate revealed over 4% variation resulting in separate phylogenetic clusters (Fig. 2). These findings suggest the circulation of genetically-distinct WNV strains in Anatolia, which could not be identified previously, probably due to the analysis of partial E gene sequences [15]. The recent report of lineage two sequences characterised from the brain tissue of a sick mare from the Marmara region provides further evidence for multiple WNV variants circulating in Anatolia, despite the availability of very limited sequence data [33]. A similar observation has also been reported from Israel [34]. Comparison of full or near full-length genomes is required for a thorough understanding of WNV genomic variability in Turkey. Unfortunately, our attempt for isolating the WNV strain using C6/36 and Vero cells have not been successful in this study, which has to be undertaken in follow-up efforts.

Sequences belonging to various ISFs have further been characterised in the mosquito pools (Table 2). Our previous efforts confined to the Thrace region and using a different screening assay had failed to identify any related strains [11]. In this study, ISFs were detected in pools originating from both the Aegean and Thrace regions, where specimens from Thrace represented the vast majority in the study cohort (Table 1). Pairwise comparisons, nucleotide and amino acid phylogenies of the obtained sequences revealed at least three distinct ISFs in circulation. Sequences closely related to CTFVs or Mediterranean *Culex* flaviviruses were recovered in 6 pools (T1, T2, T3, T4, T9 and T10) comprising *Cx. theileri* and *Cx. pipiens* (*s.l.*) specimens (Table 2). CFTV was originally isolated and characterised in detail from *Cx. theileri* mosquitoes collected in Portugal in 2009 and 2010 [35]. Evidence for the circulation of several related and potentially identical strains have been revealed [7], which include partial sequences from Spain and Portugal with different names (*Mediterranean Culex flavivirus* and *Spanish Culex flavivirus*) [8, 18]. These sequences are 91–100% identical, and comparisons with the sequences in this study reveal a maximum divergence of 11.9%. It is suggested that variation rates over 16% at the nucleotide level is required to establish any particular strain as a separate species in the family *Flaviviridae* [36]. Therefore, these sequences can be considered to represent the same *Flavivirus* species, until proven otherwise by genomic sequencing or relevant biological properties [7]. We have accomplished virus isolation in T1, T2, T3 and T9 pools and have reported the complete (three isolates) or near complete (one isolate) genome sequences of these strains previously [25]. All strains displayed a very high genetic similarity, with over 99% identity match on nucleotide and amino acid alignments, revealing them to be different isolates of the same virus, with the closest relative being the CTFV strains isolated in Portugal [25]. So far, CTFVs and related sequences are detected in *Culex* mosquitoes, including *Cx. theileri* and *Cx. pipiens* (*s.l.*) [7, 8]. Virus isolation could not be achieved in pools T4 and T10, with *Cx. pipiens* (*s.l.*) specimens or with relatively lower number of individuals (Table 2). It remains to be determined whether related but distinct ISFs are present in these pools.

Another ISF was detected in 2 pools (T6 and T7) comprising *Ae. caspius* mosquitoes (Table 2). The partial sequences are observed to be related to OCFV, HANKV and *Mediterranean Ochlerotatus flavivirus* (Figs. 3 and 4), which have also been detected in various mosquitoes from Portugal, Spain, Italy [7, 18, 26], and Greece [37]. These viruses, along with the related sequences characterised in this study, can also be considered to represent local variants of the same virus, according to the criterion explained above. Upcoming official reports on taxonomy are required to resolve the nomenclature complexities currently observed in ISFs.

We could isolate and fully characterise the strain in pool T6 in C6/36 cells. In this report, we called the strain OCFVt and used this acronym for practical purposes, to indicate the strain detected in Turkey. Since data from a single strain is available and the extreme 5' and 3' ends of the isolated strain has not been confirmed by conventional methods, we consider the current OCFVt genome as near-complete. Nevertheless, the sequence is sufficient for the detailed analyses and to infer phylogenetic relations and represents the third near-complete sequence available for similar strains. Several conserved flaviviral motifs involved in viral replication have also been identified in the OCFVt polyprotein. Genome-wide and protein-specific comparisons and phylogenetic analyses further confirmed the close relationship of OCFVt with OCFV/HANKV, with identical predicted mature viral proteins and significant nucleotide/amino acid similarities (Table 3; Additional file 1: Table S1; Fig. 5). Furthermore, OCFVt, OCFV and HANKV share certain structural properties such as predicted protease cleavage sites, the ribosomal frameshifting site followed by a stem-loop structure and the fusion peptide motif [7, 24, 25]. OCFV, OCFVt, HANKV and related sequences have not only been detected in *Aedes* mosquitoes (*Ae. caspius*, *Ae. detritus* and *Ae. vexans*), but also in *Culex* species, including *Cx. theileri* and *Cx. pipiens* (*s.l.*) [7, 18, 24, 25], a trait shared by certain ISFs [8].

We have characterised two additional ISF sequences (T5 and T11) from *Ur. unguiculata* and *Cs. annulata* mosquitoes (Table 2). T11 is observed to be closely related to two *Flavivirus* AV-2011/Spain sequences, with which it clustered in maximum likelihood analyses. Interestingly, these sequences represent a group of the ISF DNA forms, characterised in *Ae. vexans* pools from Spain [18]. The presence of DNA forms in infected cells and chromosomal integration are unique features of ISFs, observed to occur naturally in some strains [7, 8], as well as in laboratory conditions [38]. *Flavivirus* AV-2011/Spain sequences have been characterised as two distinct DNA forms in mosquito pools [18]. In addition to the group related to the T11 sequence, a second group, significantly divergent from the first with several stop codons in their sequences, have been identified, in *Ae. caspius*, *Ae. caspius* and *Cs. annulata* mosquitoes [18]. Viral RNAs or genetically-similar viral sequences were not detected. Thus, they were considered as remnant DNA forms of unidentified ISFs. The detection and characterisation of RNAs suggest the existence of a replication-competent and genetically-related ISF, a novel finding of this study. Despite our efforts, we failed to amplify DNA forms in any of the pools we had evaluated in the screening assay. However, substitutions affecting primer bindings sites have not been deeply investigated and must be considered in future efforts. Virus isolation attempts were not successful for T5 or T11, both of which included a limited number of specimens (Table 2). The infecting ISF in pool T5 could not be precisely determined with the available data.

## Conclusion

During extensive biosurveillance in three major regions of Turkey in 2014 and 2015, we have detected the circulation of WNV as well as several distinct ISFs in mosquitoes. Novel WNV variants were noted in Turkey. A virus strain of *Ochlerotatus caspius flavivirus*, tentatively named as OCFVt, was isolated and characterised. Moreover, flaviviral RNAs closely-related to an ISF previously reported in DNA form were detected suggesting the existence of a replication-competent virus.

## Additional files

Additional file 1: Table S1. Pairwise comparison of the nucleotide and putative amino acid (in parentheses) sequences of the ISF polyprotein (PDF 58 kb)
Additional file 2: Table S2. Comparison of the amino acid substitutions observed in the viral polyprotein (PDF 81 kb)

## Abbreviations

C-AC: virion C-anchored C
CTFV: *Culex theileri flavivirus*
DENV: Dengue virus
E: Envelope
HANKV: Hanko virus
ISF: Insect-specific flavivirus
JEV: Japanese encephalitis virus
MIR: Minimal infection rate
NS: Non-structural
OCFV: *Ochlerotatus caspius flavivirus*
OCFVt: *Ochlerotatus caspius flavivirus* Turkey
PARV: Parramatta River virus
PrM-M: Premembrane-membrane
RT: rReverse transcription
TBEV: Tick-borne encephalitis virus
YFV: Yellow fever virus
WNV: West Nile virus
ZIKV: Zika virus
