# Supplementary material for: Co-circulation of West Nile virus and distinct insect-specific flaviviruses in Turkey
Source: Parasit Vectors. 2017 Mar 20;10:149. doi: 10.1186/s13071-017-2087-7 (PMC5360070; doi:10.1186/s13071-017-2087-7)
Supplement: Additional file 1: Table S1. — Pairwise comparison of the nucleotide and putative amino acid (in parentheses) sequences of the ISF polyprotein (PDF 58 kb) [file 13071_2017_2087_MOESM1_ESM.pdf]

Additional file 1: Pairwise comparison of the nucleotide and putative amino acid (in parantheses) sequences of the ISF polyprotein. OCFVt: Ochlerotatus caspius flavivirus Turkey (KY345399); OCFV: Ochlerotatus caspius flavivirus (HF548540); HANKV:Hanko virus (JQ268258); PARV: Parramatta River virus (KT192549). Similarity values were indicated as percent.

	**OCFVt**	**OCFV**	**HANKV**	**PARV**
**OCFVt**	**-**	94.7 (98.6)	89 (97.1)	67.3 (71.9)
**OCFV**	94.7 (98.6)	-	88.7 (96.7)	67.6 (71.9)
**HANKV**	89 (97.1)	88.7 (96.7)	-	67.5 (77.2)
**PARV**	67.3 (71.9)	67.6 (71.9)	67.5 (77.2)	-
