# Supplementary material for: Co-circulation of West Nile virus and distinct insect-specific flaviviruses in Turkey
Source: Parasit Vectors. 2017 Mar 20;10:149. doi: 10.1186/s13071-017-2087-7 (PMC5360070; doi:10.1186/s13071-017-2087-7)
Supplement: Additional file 2: Table S2. — Comparison of the amino acid substitutions observed in the viral polyprotein (PDF 81 kb) [file 13071_2017_2087_MOESM2_ESM.pdf]

Additional file 2: Comparison of the amino acid substitutions observed in the viral polyprotein (OCFVt: Ochlerotatus caspius flavivirus Turkey (KY345399), OCFV: Ochlerotatus caspius flavivirus (HF548540), HANKV: Hanko virus (JQ268258)).

**Region**	**Position**	**Strain**		
		OCFVt	OCFV	HANKV
C	4	W	L	L
	27	T	T	A
	37	P	P	S
	44	K	R	K
	45	T	T	V
	51	V	V	A
	53	K	K	R
	54	G	S	V
	62	W	C	W
	89	L	L	F
	93	M	L	M
Anchor C	127	M	V	M
	136	S	S	T
PrM	166	I	I	T
	174	L	L	I
	175	A	A	V
	179	I	I	V
	213	V	V	I
	215	G	E	E
M	230	K	K	R
	245	L	L	M
	258	L	L	I
E	313	I	I	V
	332	G	S	S
	360	N	D	D
	411	G	G	N
	413	S	S	A
	414	V	I	I
	470	A	A	S
	500	K	Q	K
	509	V	V	I
	523	K	K	N
	551	S	S	A
	598	S	S	T
	611	I	V	V
	629	V	M	V
	645	V	M	V
	660	I	I	V
	664	I	I	F
	667	I	I	L
	691	A	A	V
	700	T	T	I
	705	I	I	M
NS1	740	A	T	T
	792	V	V	I
	811	R	R	K
	847	D	D	E
	852	I	I	V
	861	N	N	T
	921	I	I	M
	942	A	A	D

	963	N	S	N
	1075	A	T	A
	1104	R	K	R
NS2a	1108	A	A	T
	1148	R	R	K
	1203	L	M	L
	1209	G	G	S
	1218	K	K	R
	1219	L	L	S
	1297	M	M	T
	1298	Y	Y	F
	1300	T	T	M
NS2b	1338	V	V	A
NS3	1492	N	N	S
	1596	P	S	P
	1689	R	R	K
	1695	V	V	L
	1696	I	I	V
	1724	S	T	S
	1754	Q	Q	H
	1820	R	R	K
	1849	Q	H	H
	1904	D	E	E
	1908	I	I	T
	1930	S	S	H
	1933	R	R	K
	2000	V	V	I
	2012	A	A	T
	2038	S	S	A
	2047	I	I	V
NS4a	2074	P	S	S
	2079	S	S	Y
	2083	T	T	S
	2125	V	V	I
NS4b	2133	I	V	V
	2140	A	A	G
	2248	A	A	S
	2250	R	R	G
	2253	R	K	R
	2259	F	F	S
	2260	Q	R	R
	2270	F	F	M
	2292	M	V	M
	2352	I	V	I
	2359	M	M	T
	2385	A	V	T
	2402	S	S	N
	2425	V	I	V
	2436	T	A	T
NS5	2516	E	E	D
	2519	A	A	S
	2587	T	T	S
	2615	A	A	V
	2649	S	S	T
	2733	R	R	K
	2811	W	R	W
	2813	W	G	W
	2814	E	G	E
	2815	M	L	L
	2853	A	T	T

2864	R	Q	R
2867	L	L	T
2869	R	K	R
2883	E	E	D
2991	V	I	V
3050	I	I	V
3122	V	A	T
3126	N	N	D
3193	L	P	P
3350	K	-	R
3370	D	-	E
